# A comparative analysis of the heterotrimeric G-protein Gα, Gβ and Gγ subunits in the wheat pathogen *Stagonospora nodorum*

**DOI:** 10.1186/1471-2180-12-131

**Published:** 2012-07-03

**Authors:** Joel P A Gummer, Robert D Trengove, Richard P Oliver, Peter S Solomon

**Affiliations:** 1 Separation Science Laboratory, Murdoch University, Perth, 6150, WA, Australia; 2 Metabolomics Australia, Murdoch University, Perth, 6150, WA, Australia; 3 Australian Centre for Necrotrophic Fungal Pathogens, Curtin University, Bentley, 6102, WA, Australia; 4 Plant Science Division, Research School of Biology, The Australian National University, Acton, ACT, 0200, Australia

**Keywords:** G-protein, cAMP-independent signal transduction, Fungal wheat pathogen, Asexual sporulation

## Abstract

**Background:**

It has been well established that the Gα subunit of the heterotrimeric G-protein in the wheat pathogen *Stagonospora nodorum* is required for a variety of phenotypes including pathogenicity, melanisation and asexual differentiation. The roles though of the Gγ and Gβ subunits though were unclear. The objective of this study was to identify and understand the role of these subunits and assess their requirement for pathogenicity and development.

**Results:**

G-protein Gγ and Gβ subunits, named *Gga1* and *Gba1* respectively, were identified in the *Stagonospora nodorum* genome by comparative analysis with known fungal orthologues. A reverse genetics technique was used to study the role of these and revealed that the mutant strains displayed altered *in vitro* growth including a differential response to a variety of exogenous carbon sources. Pathogenicity assays showed that *Stagonospora nodorum* strains lacking *Gba1* were essentially non-pathogenic whilst *Gga1-*impaired strains displayed significantly slower growth *in planta*. Subsequent sporulation assays showed that like the previously described Gα subunit mutants, both *Gba1* and *Gga1* were required for asexual sporulation with neither mutant strain being able to differentiate either pycnidia nor pycnidiospores under normal growth conditions. Continued incubation at 4°C was found to complement the mutation in each of the G-protein subunits with nearly wild-type levels of pycnidia recovered.

**Conclusion:**

This study provides further evidence on the significance of cAMP-dependent signal transduction for many aspects of fungal development and pathogenicity. The observation that cold temperatures can complement the G-protein sporulation defect now provides an ideal tool by which asexual differentiation can now be dissected.

## Background

*Stagonospora* (Teleomorph: *Phaeosphaeria) nodorum* is a necrotrophic fungal pathogen and the causal agent of stagonospora nodorum blotch (SNB) of wheat [1]. Recent studies focused on understanding the molecular basis of the disease has identified the required role of secreted necrotrophic effectors during infection [2]. The interaction of these secreted effector proteins with a corresponding host dominant susceptibility gene results in rapid cell death and the facilitation of a rapid vegetative growth phase *in planta*.

Whilst the role of the effector proteins in causing disease is clear, it has also been demonstrated that the ability of the pathogen to undergo asexual sporulation is critical for disease progression throughout the growing season [1]. The asexual spores (pycnidiospores) of *S. nodorum* are formed in asexual structures known as pycnidia [3]. The pycnidiospores are released from the mature pycnidia on the leaf surface by rain splash dispersal leading to new infections on younger leaves. These multiple rounds of successive inoculation by the fungus, and in an inoculum density dependent manner escalates the damaging symptoms of SNB, spreading the disease to the head of the plant.

Recognition of the host by the fungus, followed by its capacity to penetrate the leaf, proliferate and reproduce is likely to require a perception of a range of signals from the host and environment, ultimately influencing disease severity. As such, heterotrimeric G-protein signalling has been the subject of intense research in filamentous fungi and many other biological systems [4].

The *Neurospora crassa Gna1* and *Gna2* genes were the first reported genes of a G-protein subunit to be cloned in a filamentous fungus [5]. In filamentous fungi, the resulting phenotypic effects of loss and gain of function mutations of the genes encoding the Gα, Gβ and Gγ proteins comprising the heterotrimer, have identified a number of cellular processes under the regulation of the G-protein. Among others, a commonly described attribute of fungal G-protein-compromised mutants is an effect on sporulation with reports of hyper-sporulation [6], reduced sporulation [7,8] or a complete lack of sporulation [9] across genera.

Reverse genetics studies in *S. nodorum* have demonstrated the requirement of several signalling pathways for disease development. Strains of *S. nodorum* lacking the Gα subunit *Gna1*[9], the mitogen-activated protein kinase *Mak2*[10], a Ca^2+^/calmodulum-dependent protein kinase *CpkA*[11], or the short-chain dehydrogenase *Sch1*[12] all demonstrate a variety of developmental defects including being either severely compromised in sporulation or are unable to do so.

Here, we report the comparison of three mutant strains of *S. nodorum* with the wild-type strain SN15. All three mutants were compromised in G-protein signalling, with each lacking one of the subunits of the heterotrimer. The *Gba1* (Gβ) and *Gga1* (Gγ)-lacking strains of *S. nodorum*, given the strain names *gba1-6* and *gga1-25*, respectively, were created by homologous recombination of the *Gba1* and *Gga1* genes with a selectable marker. The phenotypic characteristics were then assessed alongside those of the previously described *S. nodorum gna1-35* (*Gna1* mutant) strain. Consistent with *gna1-35*, the *gba1-6* and *gga1-25* strains were less pathogenic on wheat and unable to sporulate asexually. Interestingly, it was found that prolonged incubation of mature plate cultures of *gna1-35*, *gba1-6* and *gga1-25* at 4°C would complement the sporulation defect; developing pycnidia and restoring asexual sporulation in these strains. These strains are now helping aid in dissecting the molecular mechanisms underlying the phenotypic defects with the aim of bringing to light better mechanisms of controlling *S. nodorum* and other fungal pathogens.

## Results

### Identification and disruption of *Gga1* and *Gba1* in *S. nodorum*

The genes encoding putative Gγ and Gβ subunits were identified in the *S. nodorum* genome sequence by blast analysis using related fungal homologues. Using this approach, the genes SNOG_16044 and SNOG_00288 were identified as encoding putative Gγ and Gβ subunits and named *Gba1* and *Gga1* respectively. As anticipated, BlastP of both Gba1 and Gga1 revealed multiple near identical proteins in closely related fungi. A clustal analysis of these related sequences is shown in Additional file 1: Figure S1.

To investigate the role of the genes in growth and pathogenicity of *S. nodorum*, *Gga1* and *Gba1* were disrupted via homologous recombination as described above. The fungal colonies resulting from both transformations were screened by PCR to confirm homologous recombination ( Additional file 1: Figure S2). A number of successful mutations were confirmed for both the *Gga1* and *Gba1* gene disruptions. The putative mutants were selected for copy number determination as described above. All transformants demonstrated by PCR to have undergone homologous recombination had a calculated ratio of the phleomycin resistance gene to single-copy actin gene of between 0.9 and 1.1 indicating that only one copy of the transformation cassette had integrated into the genome. Representative strains for each mutation were chosen for further analysis.

### All three G-protein subunits are required for normal growth

**Figure 1:**
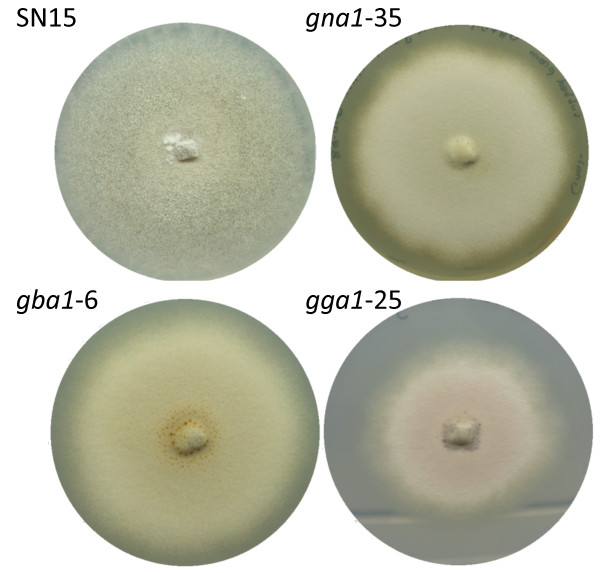
*** S. nodorum* SN15 readily develops pycnidia and asexually sporulates when cultured on minimal medium at 22°C.** Under the same culture conditions, *S. nodorum* mutant strains *gna1-35*, *gba1-6* and *gga1-25* do not develop pycnidia or sporulate and grow with a uniform ‘dry-mass’ phenotype. Minimal media was used for these experiments.

The phenotypic characteristics of the *S. nodorum gga1* and *gba1* strains were assessed *in vitro*. The previously published Gα mutant, *gna1-35*, was also included for a comprehensive analysis for the each of the three G-protein subunits. The mutant strains *gba1-6* and *gga1-25* showed a number of phenotypic effects consistent with those described for *gna1* by [9]. All three strains were non-sporulating under the standard *in vitro* culture conditions used to promote asexual sporulation in wild-type SN15. On V8PDA medium, each strain displayed pale pink mycelia, often developing a green colouration towards the centre of the culture. As the strains matured, the mycelia lost the pink and green colouration, becoming white, to display an albino phenotype. On minimal medium containing 25 mM glucose as the sole carbon source, *gga1-25* displayed a similar pink colouration, however *gna1-35* and *gba1-6* both grew albino (Figure 1). 

**Figure 2:**
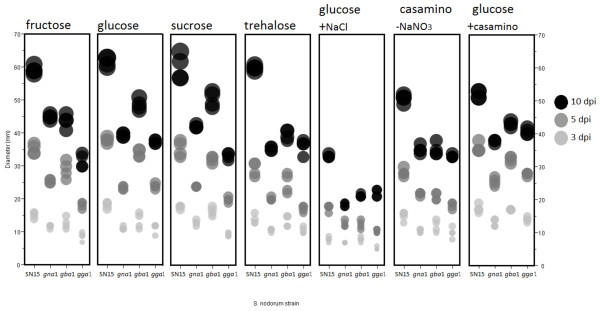
** The growth rate and phenotypic characteristics of the *S. nodorum* strains depend on the available carbon source.** Individual colony diameters (mm) are displayed as a representation of growth 3, 5 and 10 dpi with *S. nodorum* SN15, *gna1-35*, *gba1-6* or *gga1-25*. The displayed circle diameters are proportionate to colony diameter.

All mutant strains were found to have reduced radial growth by comparison to wild type, regardless of the carbon source (Figures 1 and 2, Table 1). Differences in the radial growth rate between the mutant strains however were found to be dependent on the available carbon source. *S. nodorum gba1-6* showed significantly (p < 0.05) higher radial growth than the other two mutants when provided with arabinose, glucose or sucrose. When provided with fructose however, *gba1-6* growth was significantly reduced compared to that on glucose or sucrose. *Gna1-35* growth significantly increased compared to most other carbon sources tested, such that when grown on fructose, there was no significant difference in radial growth between *gna1-35* and *gba1-6*. When *gba1-6* was provided with arabinose, although growth was equivalent to that measured on fructose, it still retained a higher radial growth than *gna1-35* as it does not have the measured increase in growth rate in response to arabinose as it does with fructose. It is evident from this data that fructose resulted in the greatest radial growth for *S. nodorum gna1-35*, whereas glucose and sucrose resulted in the greatest radial growth for *S. nodorum gba1-6.S. nodorum gga1-25* showed significantly less radial growth than all other strains on most carbon sources. On glucose *gga1-25* has a radial growth equivalent to that of *gna1-35*, and on trehalose the growth was equivalent to both *gna1-35* and *gba1-6*. When casamino acids were added along with glucose, *gga1-25* achieved its highest recorded radial growth, which was equivalent to that of *gna1-35* and *gba1-6* on the same medium (Figure 2; Table 1).

**Table 1:** Mean diameter (± standard deviation)* and evidence of pigment secretion of*S. nodorum *strains growing in the dark on minimal medium supplemented with different carbon sources

**Media supplement**	***S. nodorum*strain**	**Diameter**^**1**^**(mm)**	**Colour of secretion**
Arabinose	SN15	61.5 ± 2.4 (A)	NA
	*gna*1	36.2 ± 1.0 (CD)	NA
	*gba*1	45.5 ± 0.6 (BC)	yellow
	*gga*A	25.5 ± 1.0 (E)	NA
Fructose	SN15	59.2 ± 1.3 (A)	NA
	*gna*1	45 ± 0.8 (BC)	NA
	*gba*1	43.7 ± 2.1 (BC)	NA
	*gga*A	31.7 ± 2.1 (D)	NA
Glucose	SN15	61.7 ± 1.5 (A)	NA
	*gna*1	39.5 ± 0.6 (C)	deep orange
	*gba*1	48.7 ± 1.7 (B)	dark brown
	*gga*A	37.5 ± 0.6 (C)	light brown
Lactose	SN15	52.5 ± 1.3 (B)	NA
	*gna*1	36.7 ± 1.0 (CD)	NA
	*gba*1	40.2 ± 0.5 (C)	yellow
	*gga*A	31.0 ± 1.2 (D)	NA
Mannitol	SN15	51.0 ± 1.8 (B)	NA
	*gna*1	38.0 ± 0 (C)	NA
	*gba*1	39.2 ± 1.0 (C)	yellow
	*gga*A	28.7 ± 1.0 (D)	NA
**Media supplement**	***S. nodorum*strain**	**Diameter (mm)**	**Colour of secretion**
Sucrose	SN15	60.2 ± 3.9 (A)	NA
	*gna*1	42.2 ± 0.5 (C)	NA
	*gba*1	50.5 ± 2.4 (B)	NA
	*gga*A	33.2 ± 1.0 (D)	NA
Trehalose	SN15	60 ± 0.8 (A)	NA
	*gna*1	35.5 ± 0.6 (CD)	NA
	*gba*1	39.7 ± 1.5 (C)	NA
	*gga*A	36.2 ± 2.2 (CD)	NA
Glucose + NaCl	SN15	33.2 ± 0.5 (D)	NA
	*gna*1	18.5 ± 0.6 (E)	dark brown
	*gba*1	21.2 ± 0.5 (E)	NA
	*gga*A	22.0 ± 1.2 (E)	NA
Casamino - NaNO_3_	SN15	50.7 ± 1.3 (B)	NA
	*gna*1	35.2 ± 1.3 (CD)	NA
	*gba*1	35.2 ± 1.9 (CD)	NA
	*gga*A	33.2 ± 0.5 (D)	NA
Glucose + Casamino	SN15	52.0 ± 1.2 (B)	orange/brown
	*gna*1	37.7 ± 0.5 (CD)	orange/brown
	*gba*1	43.0 ± 0.8 (BC)	orange/brown
	*gga*A	40.7 ± 1.0 (C)	orange/brown

Wild-type SN15 and the mutant strains *gna1-35*, *gba1-6* and *ggaA-25* display carbon source-dependent changes in radial growth rate and pigment secretion as observed 10 days post inoculation (dpi).

^1^The letters in brackets shown after the diameter measurements define the significance of the results with growth assays showing the same letter being not significantly different (p < 0.05).

**Figure 3:**
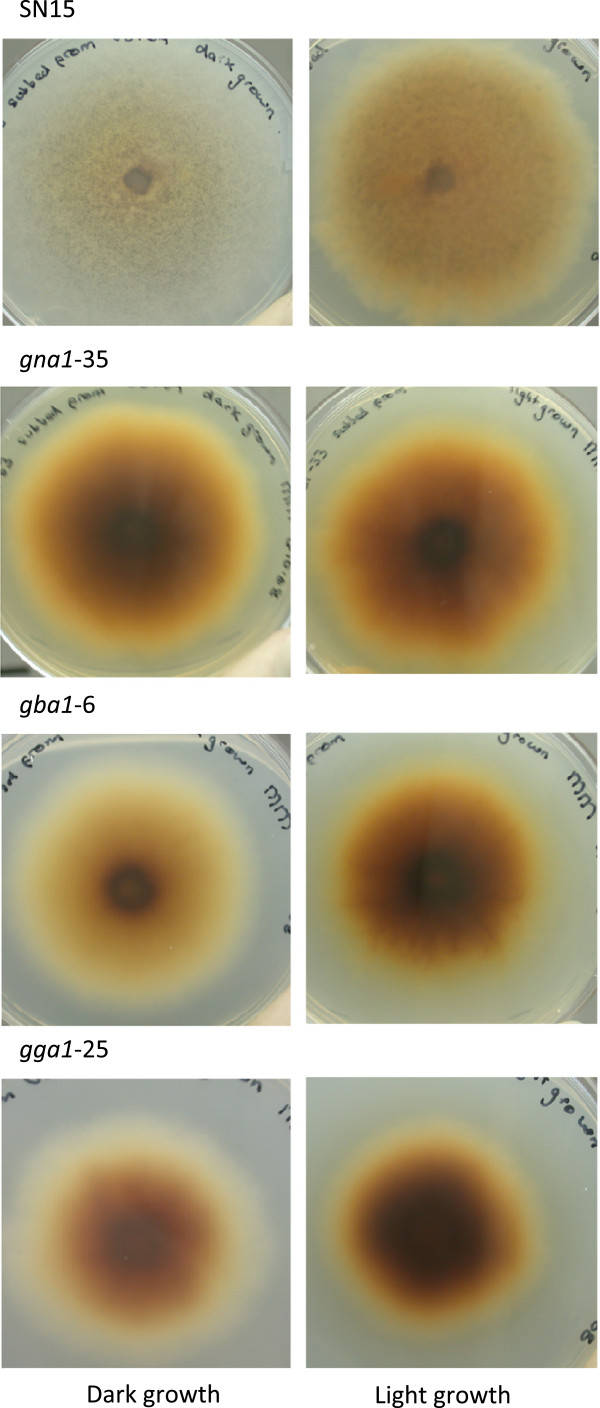
** A visual comparison of pigments secreted into the medium by strains of *S. nodorum * when grown in the dark, compared to those grown under a 12 hour white light cycle.** Discolouration of the medium is dramatically intensified in cultures of *S. nodorum* wild-type SN15 when exposed to light; less so for mutant strains *gba1-6* and *gga1-25*; with little change between the light and dark cultured *gna1-35* mutant. Agar cultures are pictured from beneath the petri-dish.

It has been previously observed that wildtype *S. nodorum* secretes a light brown coloured pigment when grown on minimal medium (25 mM glucose) under white light (Figure 3) [9]. By comparison *gna1-35* secretes a much darker coloured pigment, with little difference between light and dark grown cultures. Under these conditions, cultures grown in the light showed a pronounced medium discolouration, whilst *gba1-6* and *gga1-25* both routinely showed pigment secretion when grown in the light and dark. Pigment secretion, as observed by the intensity of the discolouration of the growth medium, was also dependant on the carbon source (Table 1). Whilst *gna1-35* had the most pronounced pigment secretion of the strains when grown on glucose, this strain did not show any notable discolouration on any of the other carbon sources tested, nor did *gga1-25*. On glucose, *S. nodorum gba1-6* secreted pigments when the medium was supplemented with lactose, mannitol or arabinose. However, when provided with fructose, sucrose or trehalose, no pigment secretion was noted for this or any other strain. 

### *Gna1*, *Gba1* and *Gga1* are all required for different aspects of pathogenicity on wheat

**Figure 4:**
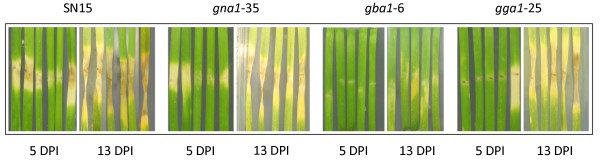
** Detached leaf assay (DLA) of wheat leaf (cv. Calingiri) inoculated with *S. nodorum* wild-type strain SN15 and mutant strains *gna1-35*, *gba1-6* and *gga1-25*, displayed at 5 and 13 DPI.**

Detached leaf assays (DLAs) were used to compare the differences in pathogenicity of *S. nodorum* strains on wheat. Figure 4 shows the slowed progression of lesion formation by the mutant strains on wheat compared to the wildtype. After 5 dpi, SN15 causes necrotic flecking of the leaf, whilst the mutant strain *gna1-35* produced a chlorotic lesion. The *gba1-6* and *gga1-25* strain only showed very mild chlorosis on most leaf replicates at the same time after inoculation. The same leaves at 13 dpi infected with *gna1-35* or *gga1-25* exhibit disease symptoms comparable to those produced by SN15. However, given this extended timeframe disease symptoms of leaves challenged with *gba1-6* at this latter stage have not progressed beyond a very mild chlorotic response. Sporulation was not evident for any of the mutants in planta.

### Prolonged cold exposure induces pycnidia differentiation

Whilst pycnidial development and the accompanying asexual sporulation of *S. nodorum* SN15 occurs readily on agar plate media, under the same conditions, the mutant strains *gna*1-35, *gba*1-6 and *gga1*-25 as described above are completely absent of pycnidia formation.

**Figure 5:**
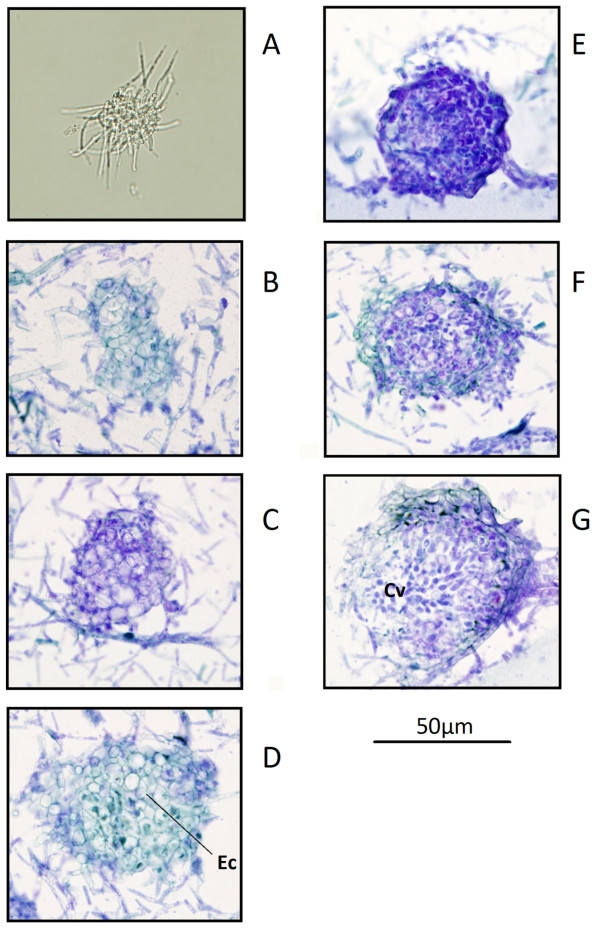
** Pycnidia development progresses slowly in the mutant *S. nodorum *strains under study.** Longitudinal sections of a wax embedded excision of a *S. nodorum gga1-*25 culture -stained with toluidine blue, is pictured. Slow differentiation of mycelia into pycnidia allowed all stages of development to be captured in an excision from a single culture. Pynidia formation begins with the intertwining of mycelia to form a mycelial knot (**A**), which is followed by differentiation and enlargement of the cells (Ec), forming a primordium (**B** through **F**), which matures into the pycnidium (**G**), eventually producing pycnidiospores from the conidiogenous cells (Cv) within the pycnidial cavity.

It was observed however that the incubation of the strains at 4°C from 8 dpi resulted in the appearance of small dark dots that resembled the initiation of asexual development. A continuation of the incubation of these cultures at the colder temperatures revealed that these conditions appeared to promote the pycnidial development. Toluidine blue stained sections of these spots identified the regions as intertwining mycelia (Figure 5). Continued incubation of G-protein mutants at the lower temperature allowed the intertwining to progress to the formation of a mycelial knot. Mycelial knot formation is the earliest stage of pycnidia formation, preceding differentiation of the mycelial cells [3]. Subsequent observation of the mycelial knot showed differentiation of the mycelia into pycnidia within four to six weeks at 4°C. This is a significant result as asexual development had not yet been observed in a *S. nodorum* G-protein signalling mutant. Similar results were also observed for the *gbaA* and *gna1* strains (data not shown). 

**Figure 6:**
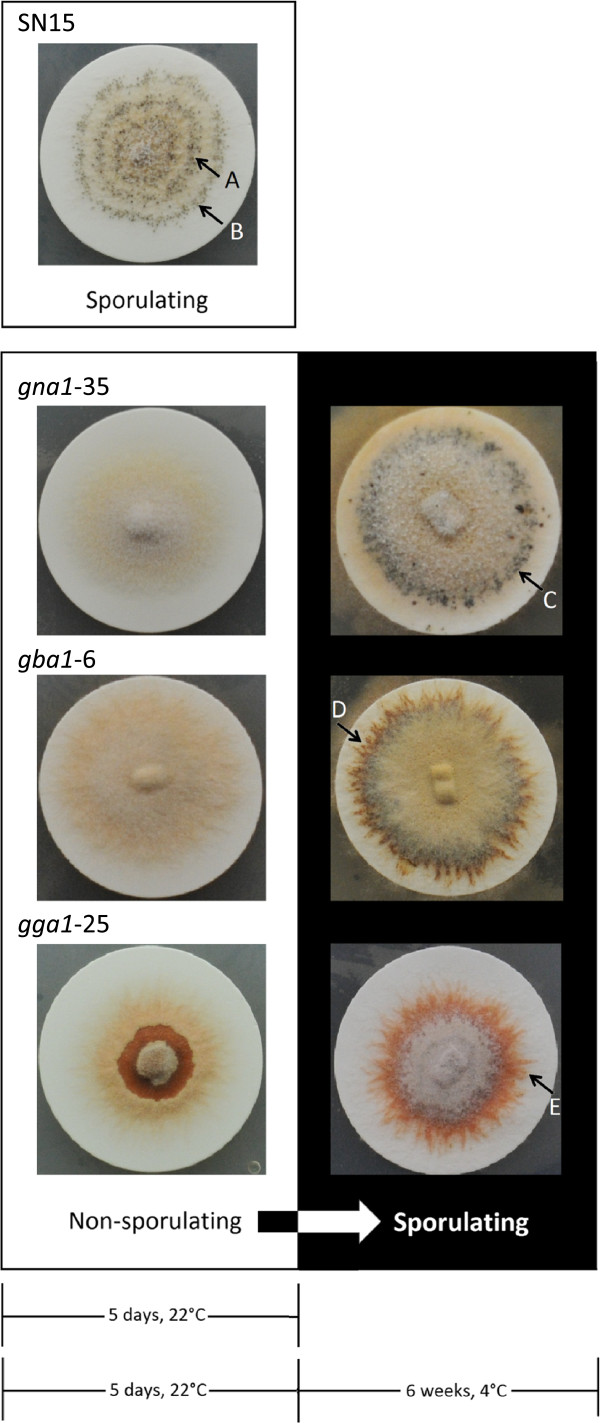
Pycnidia development (accompanied by asexual sporulation) in the *S. nodorum* wild-type strain SN15 is observed in a distinct circadian ring pattern (A and B) within 5 days post inoculation (dpi) of solid minimal medium*. Pycnidia do not develop in the mutant strains during this timeframe. The formation of pycnidia in the *S. nodorum* mutant strains *gna*1-35, *gba*1-6 and *gga1*-25 is evident amongst the outer mycelia (C - E) from between 3 and 6 weeks incubation of (the initially) non-sporulating (5 dpi) culture at 4°C. *S. nodorum* strains are pictured growing on nitrocellulose membranes (30 mm diameter)-overlaying minimal medium agar.

**Figure 7:**
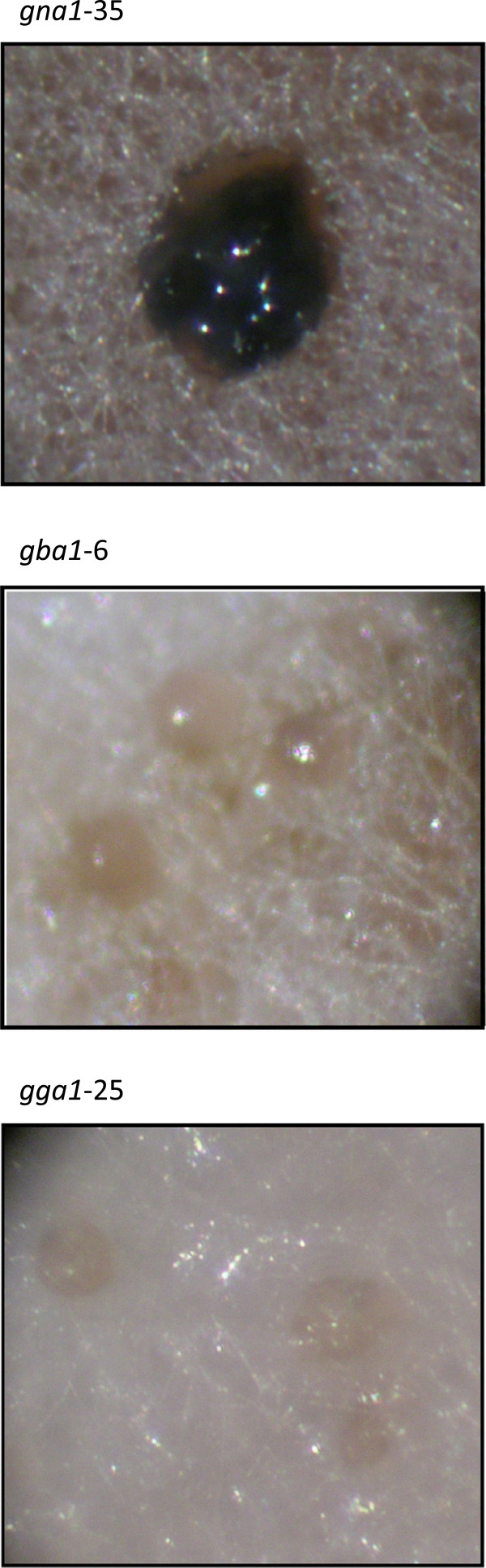
** The observed pigmentation and size of the mutant pycnidium differs significantly between strains.** Pictured is a single *gna1-35* pycnidium, and pycnidia of the *gba1-6* and *gga1-25* strains of *S. nodorum,* amongst the mycelia. Images captured at 40× magnification.

Pycnidia (accompanied by asexual spore development) in *S. nodorum* wild-type SN15 developed in a distinct circadian ring pattern within 5 days from inoculation (dpi) of solid minimal medium (Figure 6). The formation of pycnidia (containing viable spores) in *S. nodorum* mutant strains *gna1-35*, *gba1-6* and *gga1-25* by comparison was evident mainly amongst the outer perimeter of the mycelia after prolonged growth at 4°C. The pycnidia of *gna1-35* were heavily pigmented, black in appearance, (Figure 6 &7) and randomly dispersed amongst the colony’s mycelial perimeter. By comparison, *gba1-6* which developed lighter, brown-coloured pycnidia, tending to form along the mycelium as it intertwined at the perimeter of the colony. The pycnidia of *gga1-25* were comparatively lighter in colour than SN15, *gna1-35* or *gba1-6*, with a light brown-colouration, and although they often developed along the intertwining mycelium like *gba1-6*, they appeared less confined to this location of development. The pink cirrhus that exudes from pycnidia of *S. nodorum* SN15 was not evident for any of the mutant pycnidia, and perhaps consequently, spores could only be released by manual disruption. It is significant to note that though that the pycnidiospores released by the mutant were viable (Additional file 1: Figure S3).

**Figure 8:**
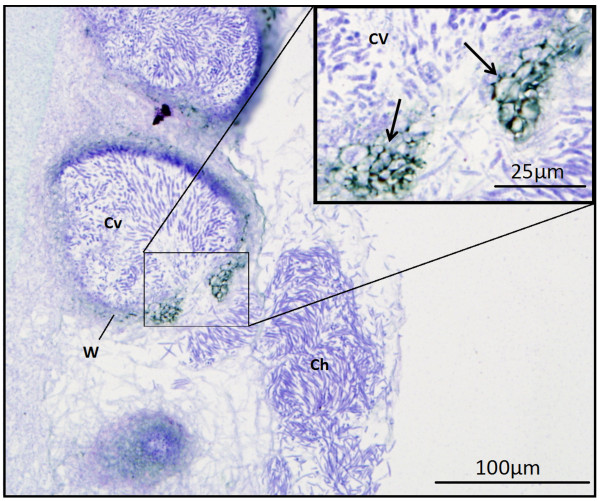
** A longitudinal section of a wax embedded excision from an asexually sporulating culture of *S. nodorum* SN15 -stained with toluidine blue.** Pictured are pycnidiospores being released from a mature pycnidium. Arrows point to the masses of enlarged cells producing the ostiole, formed in the development of the mature pycnidium, from which the pycnidiospores are released from the pycnidial cavity as a cirrus. Cv, pycnidial cavity; W, pycnidial wall; Ch, cirrus.

**Figure 9:**
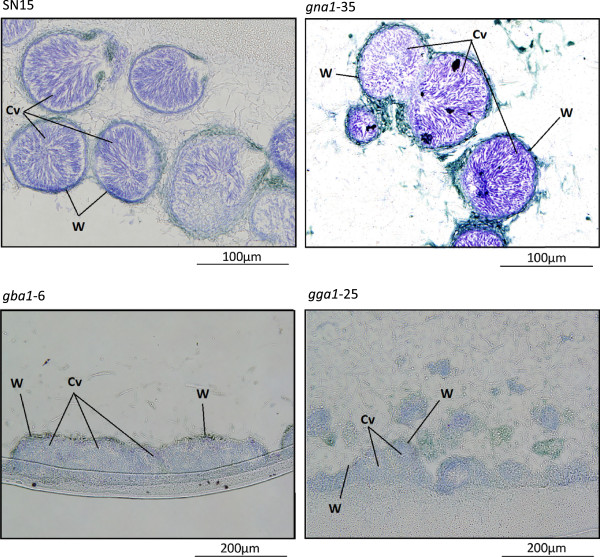
** Longitudinal sections of a wax embedded excision from asexually sporulating cultures of *S. nodorum*-stained with toluidine blue.** Pictured are conidiogenous cells and pycnidiospores contained within the mature pycnidia of wild-type strain SN15, and the (potentially less) ‘matured’ pycnidia of mutant strains *gna1-35*, *gba1-6* and *gga1-25*. The pycnidia of SN15 and *gna1-35* often develop fused, but the pycnidial cavities remain visually distinct, by comparison to those of *gba1-6* and *gga1-25* which often form a single body of pycnidia. Cv, pycnidial cavity; W, pycnidial wall.

The mature pycnidia formed under the cold-inducing conditions were visualized by light microscopy. Toluidine blue-stained longitudinal sections of *S. nodorum* SN15 pycnidia identified the presence of a number of enlarged cells that form the opening of the ostiole from which the cirrhus of spores are released from the pycnidial cavity (Figure 8). Analysis of the pycnidia formed by each of the G-protein mutants by cold induction revealed the structures to be comparable to those produced by the wildtype strain under the same conditions. It was observed that the ostiole failed to differentiate on the mutant pycnidia (Figure 9). This observation was consistent with the requirement that the pycnidiospores within these mutant pycnidia could not be released by water (as typically observed in wildtype pycnidia) and required manual disruption. The pycnidia of *gba1-6* and *gga1-25* were also nearly always observed as multiple structures fused together and were almost never seen individually (Figure 9). Although the pycnidia of SN15 and *gna1-35* often developed fused, it was uncommon for the pycnidia to form indistinct from one another. The pycnidia of *gga1*-25 and *gba*1-6 were also comparatively misshapen and less mature in appearance than those of SN15 and *gna1-35*.

## Discussion

The deactivation of the Gα subunit *Gna1* from *S. nodorum* has proven fruitful to further understanding the pathogenesis of this fungal pathogen [9]. The lack of sporulation and reduced pathogenicity of the resulting *gna1* strain sparked further investigation into the molecular and phenotypic attributes of this mutant strain largely because a determination of the molecular processes underpinning the phenotype could lead to more targeted control of the pathogen. Subsequent analysis of the gna1 strain to identify downstream-regulated targets and processes has uncovered many interesting aspects of the disease including mycotoxin production.

Based on the success of dissecting the *gna1* phenotype, we have extended this study to include the analysis of the Gβ and Gγ subunits of the heterotrimeric G-protein through a reverse genetics approach. Strains of *S. nodorum* lacking these genes displayed variety of independent phenotypes during growth *in vitro*. One of the most apparent phenotypic defects under normal growth conditions was the complete lack of pycnidia formation or accompanying asexual sporulation. This phenotype is shared by other *S. nodorum* strains possessing defects in signalling pathways, and as such, was consistent with earlier findings in *S. nodorum*[9,11,13]. Along with growth defects *in vitro*, the mutant strains also exhibited different abilities to cause disease. Lesion formation on leaves inoculated with strains lacking with *Gna1* or *Gga1* was delayed but appeared comparable to that of the wild type after two weeks post inoculation. Leaves inoculated with *Gba1* though failed to elicit any response from the leaves after 5 dpi, and only a very mild chlorotic response was evident after two weeks. This implies that *Gba1* has a critical role in disease development in *S. nodorum*. Given the almost complete lack of symptom development, it could be suggested that *Gba1*, like *StuA*[14], has a role in effector regulation. However this is only speculation and requires further analysis.

### Nutrient sensing in the *S. nodorum gna1*, *gba1* and *gga1* strains

Dramatic growth differences between the mutant strains and the wild-type SN15 were noted on agar plate medium. On V8PDA, SN15 grows radially symmetrical with pycnidia forming in distinct circadian bands [15]. The *gna1* and *gba1* mutant strains both show a similar banding pattern, in mycelial growth, indicating that these strains have not lost the capacity to perceive a light signal.

The radial growth of all three mutant strains 10 dpi was reduced by comparison to SN15 on all tested media. The variation in radial growth of the mutant strains when growing on different carbon sources confirmed that the *S. nodorum* G-protein(s) play(s) a role in carbon source utilization. In comparison to the wild-type SN15, which displayed a statistically similar radial growth rate when provided with arabinose, fructose, glucose, sucrose or trehalose as a sole carbon source.

The comparatively slower growth of *gna*1 on sucrose was interesting when considering this strain’s slower growth on glucose, but significantly higher growth on fructose. Kraakman et al., (1999) showed that the GPCR Gpr1 binds extracellular glucose in the yeast *Saccharomyces cerevisiae* and stimulates cAMP synthesis through the Gα subunit Gpa2. Likewise Lemaire et al., (2004) showed both glucose and sucrose induced cAMP signalling through the receptor Gpr1, however it was not fructose-induced. Although deletion of either *Gpr1* or *Gpa2* did not result in a reduced growth rate in *S. cerevisiae*, the strains in the study were not limited to a single carbon source [16]. However, an altered capacity to sense and respond to the presence of these sugars will inevitably affect growth. The reduced radial growth rate of the *S. nodorum gna1* mutant when solely provided with glucose or sucrose compared to fructose therefore could be due to a reduced capacity for sensing glucose and sucrose and imply similar functions for the *S. nodorum* Gna1 and yeast Gpa2. It has also been shown that in binding glucose, the GPCR Gpr1 will fail to cause the normal rapid activation of adenylate cyclase if the glucose is not internalized and phosphorylated [16], which may further explain slower growth in response to glucose in strains where the deactivated subunit causes a lesser response to glucose.

Irrespective of the speculated extracellular sensing roles of these G-protein subunits, the difference in growth rates across *S. nodorum gna1*, *gba1* and *gga1* strains when provided with these carbon sources can be explained by processes biochemically downstream. Alterations in catabolic processes may have arisen as a result of the mutations. The growth rates of *gna*1 on each of fructose and glucose, compared to sucrose, for example is consistent with processes downstream of sucrose (α-D—fructofuranosyl α-D-glucopyranoside) hydrolysis, which yields one unit of fructose and one of glucose. Given that *gna*1 grows faster on fructose, it suggests that glucose may be feeding less efficiently into glycolysis in this strain.

Interestingly the seemingly inherently slower growth rate of *S. nodorum gga1* under most conditions is comparable with each of the mutant strains when provided with trehalose as a sole carbon source. The radial growth rates on trehalose could also implicate all three subunits in processes downstream of extracellular sensing. The hydrolysis of trehalose (α-D-glucopyranosyl-α-D-glucopyranoside) yields two glucose units, yet the growth of *gba*1 is particularly slower on trehalose than glucose, which may suggest rather than a glycolytic inefficiency as mentioned above, a reduced capacity to hydrolyse trehalose, or even a diminished capacity to sense the signals that would otherwise cue the cell to catabolise trehalose. Changes to trehalose metabolism have been shown to have dramatic effects on sugar metabolism in general, and shown to have severe implications for phytopathogenicity [17,18], so the reduced capacity to use trehalose as a sole carbon source has likely had direct implications on fungal fitness.

### Metabolite secretion by the *S. nodorum gna1, gba1* and *gga1* strains

*S. nodorum gna1* has been shown to secrete brown pigments comprised of tyrosine, phenylalanine and L-DOPA into the growth medium, first observed in the discolouration of the growth medium [9]. Discolouration at the growth medium is also an attribute of *S. nodorum* SN15 and the *gba1* and *gga1* mutant strains. The carbon source dependency and intensity of discolouration of the medium also imply implications at least for primary metabolism, in the mutant strains. The carbon-source dependency of media discolouration identifies differences in metabolite secretion resulting from likely metabolic changes. These could potentially result from the inefficient use of metabolites or products of metabolism due to blockages or even over-active biochemical pathways. Together with the reduced growth rates on different media, the *Gna1*, *Gba1* and *Gga1* mutations appear to have introduced metabolic inefficiencies.

In the later observed cultures of *S. nodorum gna1*, *gba1* and *gga1*, where pycnidia formation was studied, more intense secretions could be seen. It’s likely that the intensity of media discolouration was heightened by accumulation over the extended culture period however it may also be that the secretions changed as the cultures’ phenotypes changed. It’s also possible that the increased concentration of secreted metabolites in the culture medium played a role in triggering the formation of pycnidia in these strains. Either way, the increased presence of secreted metabolites in these strains whilst undergoing pycnidial differentiation adds further interest to the identity of these secreted metabolites.

### Pathogenicity and asexual sporulation of the *S. nodorum gna1*, *gba1* and *gga1* strains

The capacity to rapidly increase fungal inoculum density by releasing spores from pycnidia following infection of the wheat plant by *S. nodorum* is fundamental to the success and consequently the impact of SNB. *S. nodorum gna1*, *gba1* and *gga1* were all unable to sporulate during infection of the wheat leaf, however although this defect may slow disease amplification, sporulation is clearly not a prerequisite for leaf necrosis. The inability for disease caused by infection with the *gba1* strain to progress beyond chlorosis however, may implicate necrotrophic effector production in *S. nodorum* as positively regulated by G-protein signalling through the Gβ subunit Gba1 [14]. It is interesting to note that the requirement of the Gβ and Gγ subunits for infection in different fungal plant pathogens varies. For example, it has been previously demonstrated that GBB1 in *Gibberella monoliformis* is not required for pathogenicity whist the orthologous protein in the related *Fusarium oxysporum* is [19,20]. Our data clearly show that gene encoding for the Gβ subunit, *Gba1*, is required for *S. nodorum* to cause disease on wheat.

Whilst sporulation was not observed for the *gna*1, *gba*1 or *gga1* strains *in planta*, the observations of asexual sporulation described *in vitro* are of considerable interest. The capacity for the *gna1*, *gba1* and *gga1* strains to develop pycnidia during prolonged incubation at 4°C from an already matured, yet non-sporulating culture adds further interest and potential for using these strains to dissect these fundamental processes in *S. nodorum*.

The physical characteristics of the mutant pycnidia observed *in vitro* were also of interest. In *S. nodorum* SN15, differentiation of cells forming the ostiole of the mature pycnidial wall was observed, but was not seen for the mutant pycnidia. It is likely that spores could not be released by the fungal strains because of this defect. Cirrus containing the spores was also observed in SN15, but not in the mutant pycnidia. Without the cirrus, it is unlikely there would be enough turgor pressure to release the spores, even with the formation of a wild-type ostiole, and it may be that this pressure plays a role in the formation of the ostiole in the *S. nodorum* pycnidium.

The pycnidia of the strains *gga1* and *gba1* are comparatively misshapen and less mature in appearance than those of SN15 and *gna1*. However, because these strains do develop viable spores, they may not actually be less mature, but perhaps this manifestation is a consequence of these two strains lacking the capacity to develop such a well-defined pycnidial wall.

In conclusion, this study has demonstrated the critical, and yet independent, roles of the heterotrimeric G-protein subunits in *S. nodorum*. Each of these subunits was found to play a role in *in vitro* and *in planta* growth, albeit with varied roles. As had been previously observed for the *gna1* strain, *gba1* and *gga1* strains were unable to sporulate when grown under normal growth conditions. However, prolonged incubation of these strains at 4°C appeared to complement the sporulation defect and pycnidia, containing viable pycnidiospores, were differentiated in each of the mutants. The mechanism of how colder temperatures induce sporulation in these mutants is clearly of interest and is the focus of ongoing studies. It should be noted that whilst single event homologous recombination events were demonstrated for each of the mutants generated in this study, future studies will attempt to complement these strains to provide unequivocal proof of the role of these in the above described phenotypes.

## Methods

### Fungal strains and media

*S. nodorum* SN15 was provided by the Department of Agriculture, Western Australia. The fungus was routinely grown on CzV8CS [45.4 g/l Czapek Dox agar (Oxoid), 10.0 g/l agar, 3.0 g/l CaCO_3_, 200 ml/l Campbell’s V8 juice, 20.0 g/l casamino acids, 20 g/l peptone, 20 g/l yeast extract, 3 g/l adenine, 0.02 g/l biotin, 0.02 g/l nicotinic acid, 0.02 g/l p-aminobenzoic acid, 0.02 g/l pyridoxine, 0.02 g/l thiamine] containing 1.5% agar. Plates were incubated at 22_C in 12 h cycles of darkness and near-UV light (Phillips TL 40 W/05). Liquid cultures were started with the addition of 10^7^spores to 100 ml CzV8CS and were grown at 22°C shaking at 130 rpm in the dark. For experiments that required defined growth conditions, *S. nodorum* SN15 was used to inoculate minimal medium (MM), which consisted of 30 g/l sucrose, 2 g/l NaNO_3_^-^, 1.0 g/l K_2_HPO_4_, 0.5 g/l KCl, 0.5 g/l MgSO_4_.7H_2_O, 0.01 g/l ZnSO_4_.7H_2_O, 0.01 g/l FeSO_4_.7H_2_O and 0.0025 g/l CuSO_4_.5H_2_O. Agarose (15 g/l) was added when plates were required.

The capacity for the *S. nodorum* strains to grow on different carbon sources was assayed by inoculation of MM agar plates containing different carbon sources by supplementing a stock of MM without the carbon source with 25 mM of the appropriate carbon source. *S. nodorum* strains were inoculated onto the above media from minimal medium (25 mM glucose) by excising a region of the agar containing approximately 4 mm^2^ of the agar surface of the (non-sporulating) growing edge of the mycelia onto the plates. Cultures were grown for 10 days in the dark at 22°C and colony diameters recorded 3, 5 and 10 days from inoculation, and observations of phenotype made. Four replicates were prepared per strain per carbon source assay. All statistical analyses were undertaken using the JMP7 package (SAS Institute). Statistical significance was determined using the Tukey–Kramer analysis.

### Plant growth conditions

Plant material and infection conditions Pots (10 cm diam.) containing Perlite (P500) and grade 2 Vermiculite (The Perlite and Vermiculite Factory, WA, Australia) were seeded with five seeds of the wheat variety Amery and grown at 20**_** C in a 12 hr day/12 hr night cycle. The pathogenicity of the mutants was assayed on detached leaves from 2-week-old wheat seedlings, using a method modified from that described by Benedikz et al. [9,21]. The distal end (2 cm) of the detached wheat leaves was removed. The next portion (4–5 cm) was embedded into benzimidazole agar, adaxial side up. The leaves were inoculated with small blocks of mycelium (approximately 45 mm^3^) and incubated in a 12 h light/12 h dark cycle at 22°C to enable disease development.

### Molecular methods

Genomic DNA (gDNA) was extracted and isolated from *S. nodorum* mycelia using a Retsch® MM301 lyser (Retsch®, UK) at 30 (Htz; 1/s) and the QIAGEN BioSprint 15 using the BioSprint 15 DNA Plant Kit protocol (QIAGEN, Australia). DNA concentrations were determined using a NanoDrop^TM^ ND-1000 (Thermo Fisher Scientific Inc., USA).

### Synthesis of the *Gga1* and *Gba1* gene disruption constructs

A construct for the disruption of *S. nodorum Gga1* was synthesized using the 5′ and 3′ UTRs flanking the putative *S. nodorum Gga1* (SNOG_00288), and the phleomycin cassette from the plasmid vector previously constructed and described by Solomon *et al.*[11]. The disruption of the *Gga1* gene was performed using a split-marker approach [11]. To create the split-marker, the phleomycin cassette was PCR amplified in two sections (with a 145 bp overlap) designated PHL and LEO -using the two PCR primer sets PHLprimer and M13R, and LEOprimer and M13F, respectively. Note that all primer sequences are listed in Additional file 2: Table S1. The two genomic UTRs flanking *Gga1* were also amplified, using the PCR primer sets *Gga1*KO5′F and *Gga1*KO5′R, and *Gga1*KO3′F and *Gga1*KO3′R. Fusion of the resulting PCR products; PHL with *Gga1*KO3′, and LEO with *Gga1*KO5′ was achieved by combining equimolar amounts (between 15 and 45 fmol) of each as template in a fusion PCR consisting of 5 μM each of PHLprimer and Gga1KO3′r, or LEOprimer and Gga1KO5′f, 1 U TaKaRa Ex Taq^TM^ DNA polymerase and 1 × TaKaRa PCR Buffer (TAKARA BIO. INC., Japan), and 10 mM dNTPs in a final reaction volume of 20 μl. Thermal cycling consisted of 94°C for 2 minutes, 35 rounds of (94°C for 15 seconds, 55°C for 15 seconds, 72°C for 2 minutes) and a final hold at 72°C for 10 minutes.

The disruption construct was developed by amplifying an 800 bp 5′ flanking region to *Gba1* using the primers GbetaKOF1 and F2 and cloning this into the *Kpn*I and *Xho*I sites in pBSK-phleo [11]. Similarly, the 823 bp 3′ flank of *Gba1* was amplified with GbetaKOR1 and R2 and cloned into *Pst*1 and *Bam*HI sites subsequent to the 5′ flank cloning. The subsequent construct, pBphleo-GβKO, was transformed into *S. nodorum* SN15 as described below.

### Preparation and transformation of *S. nodorum* protoplasts

Protoplasts were prepared from *S. nodorum* mycelia as described by Solomon *et al.*[11]. Transformation was performed as per Solomon et al. [22]. Fungal transformants were screened for homologous recombination by PCR. PCR primers were designed to anneal to the non-coding genomic regions flanking either *Gba1* or *Gga1* in *S. nodorum* SN15. The screening primers are listed in Table 1.

### RT-(q)PCR determination of gene copy number

The number of targeted gene insertions following fungal transformation was determined by quantitative real-time PCR (RT-qPCR) as described by [23]. Briefly, this involved calculating the ratio of the RT-qPCR determined cycle-threshold (CT) of the inserted phleomycin cassette to that of an endogenous single-copy actin gene; comparative to a known single-copy phleomycin cassette-possessing strain of *S. nodorum*.

CT Values were determined from reactions consisting of four gDNA amounts (100 ng, 33.5 ng, 10 ng and 3.35 ng) for each template, performed in triplicate. The primer pairs PhleoqPCRf and PhleoqPCRr or ActinqPCRf and ActinqPCRr were each used at 1.2 μM with 1× QuantiTect SYBR Green PCR Master Mix (DNA *Taq* Polymerase, QuantiTect SYBR Green PCR Buffer, dNTPs, SYBR Green I dye; Qiagen, Australia), in a reaction volume of 15 μl. Thermal cycling consisted of 95°C for 15 minutes, followed by 40 cycles of (94°C for 15 seconds, 57°C for 30 seconds and 72°C for 30 seconds).

### Histological staining and microscopy

Cross-sections of fungal tissues were examined by compound microscopy as described by [12]. An excised region of the culture was fixed overnight in FAA [3.7% (v/v) formaldehyde, 5% (v/v) glacial acetic acid, 47% (v/v) ethanol] and dehydrated in 3 hour stages of ascending concentrations of ethanol, at 70%, 90% and 100%. Cultures were then rinsed in chloroform and infiltrated with molten Paraplast® paraffin wax and the fungal culture cross-sectioned in 10 μm sections with a Shandon MX35 blade using a Leica Microtome RM225 (Leica Microsystems). Series of sections were embedded to a glass slide by overnight incubation at 60°C. Wax was removed from the sectioned tissue by two 5-minute rinses with xylene. Sections were stained with 1% toluidine blue. Light microscopy was performed using an Olympus BH-2 compound microscope equipped with Olympus DP12 image acquisition software (Olympus America Inc., USA).

## Author’s contributions

JPG carried out most of the experiments and participated in the drafting of the manuscript. RPO and RDG participated in the design of the study and the interpretation of the data. PSS conceived the study, participated in the experiments and wrote the manuscript. All authors read and approved the final manuscript.

## Supplementary Material

Additional file 1 ** Figure S1.** ClustalW alignment of *S. nodorum* (A) Gba1 and (B) GgaA with fungal orthologues. **Figure S2.** (A) Agarose gel electrophoresis of PCR products arising from the amplification of the (A) *GgaA* locus of the created *S. nodorum* mutants. Targeted Insertion of the phleomycin cassette in place of the *S. nodorum GgaA* gene results in a 4196 bp amplicon (Lanes 25, 26, 30, 31) , replacing the 1789 bp amplicon of the wild type (WT) SN15. MW, Molecular weight marker; WT, *S. nodorum* SN15 gDNA; NTC, no template PCR control; the remaining lanes labeled by mutant culture number. Lanes 1, 2, 11, 20, 32, 34, no observed amplification or (B) *Gba1* locus of strains transformed with the *Gba1* homologous disruption construct. A band of 6.1 kb represents the wildtype locus and 7.6 kb the locus having undergone homologous recombination with the disruption construct. Lane 1, 1 kb ladder; Lane 2, *S. nodorum* SN15 (wildtype); Lanes 3–8, a representative selection of transformants. Strains represented in lanes 4, 6 and 7 have all undergone homologous recombination and represent *Gba1* mutants. **Figure S3.** Light microscopy of the asexual spores of S. nodorum, harvested from the wild-type SN15 and mutant strains gna1-35, gba1-6 and ggaA-25. Click here for file

Additional file 2 ** Table S1.** Sequences of primers used in this study. Click here for file
