# Supplementary material for: A comparative analysis of the heterotrimeric G-protein Gα, Gβ and Gγ subunits in the wheat pathogen Stagonospora nodorum
Source: BMC Microbiol. 2012 Jul 3;12:131. doi: 10.1186/1471-2180-12-131 (PMC3492189; doi:10.1186/1471-2180-12-131)
Supplement: Additional file 1 — Figure S1. ClustalW alignment of S. nodorum (A) Gba1 and (B) GgaA with fungal orthologues. Figure S2. (A) Agarose gel electrophoresis of PCR products arising from the amplification of the (A) GgaA locus of the created S. nodorum mutants. Targeted Insertion of the phleomycin cassette in place of the S. nodorum GgaA gene results in a 4196 bp amplicon (Lanes 25, 26, 30, 31) , replacing the 1789 bp amplicon of the wild type (WT) SN15. MW, Molecular weight marker; WT, S. nodorum SN15 gDNA; NTC, no template PCR control; the remaining lanes labeled by mutant culture number. Lanes 1, 2, 11, 20, 32, 34, no observed amplification or (B) Gba1 locus of strains transformed with the Gba1 homologous disruption construct. A band of 6.1 kb represents the wildtype locus and 7.6 kb the locus having undergone homologous recombination with the disruption construct. Lane 1, 1 kb ladder; Lane 2, S. nodorum SN15 (wildtype); Lanes 3–8, a representative selection of transformants. Strains represented in lanes 4, 6 and 7 have all undergone homologous recombination and represent Gba1 mutants. Figure S3. Light microscopy of the asexual spores of S. nodorum, harvested from the wild-type SN15 and mutant strains gna1-35, gba1-6 and ggaA-25. [file 1471-2180-12-131-S1.pdf]

(A)

```
S. nodorum                        MADMNQESIQQKIQIARRDAEALKDRIKRKKDELADTTLRDVARDRVEALPRLTMKTKRT
Cochliobolus heterostrophus       MADMNQETIQQKIQLARRDAEALKDRIKRKKDELADTTLRDVARDRVEALPRLTMKTKRT
Leptosphaeria maculans            MADMNQESIQQKIQIARRDAEALKDRIKRKKDELADTTLRDVARDRVEALPRLTMKTKRT
Pyrenophora teres f.sp. teres     ---MNQETIQQKIQTARRDAENLKDKIKRKKDELADTTLRDVARDRVEALPRLTMKTKRT
Mycosphaerella graminciola        MADQN-ESVQQRIQIARRDAEALKDRIKRKKDELADTTLRDVARQRTEQLPRLAMKTKRT
                                      * *  ** ** ****** *** ***************** * * **** ******

S. nodorum                        LKGHLAKIYAMHWSTDRRHLVSASQDGKLIIWDAYTTNKVHAIPLRSSWVMTCAYSPSGN
Cochliobolus heterostrophus       LKGHLAKIYAMHWSTDRRHLVSASQDGKLIIWDAYTTNKVHAIPLRSSWVMTCAYSPSGN
Leptosphaeria maculans            LKGHLAKIYAMHWSTDRRHLVSASQDGKLIIWDAYTTNKVHAIPLRSSWVMTCAYSPSGN
Pyrenophora teres f.sp. teres     LKGHLAKIYAMHWSTDRRHLVSASQDGKLIIWDAYTTNKVHAIPLRSSWVMTCAYSPSGN
Mycosphaerella graminciola        LKGHLAKIYAMHWSTDRRHLVSASQDGKLIIWDAYTTNKVHAIPLRSSWVMTCAYSPSGN
                                  ************************************************************

S. nodorum                        YVACGGLDNICSIYNLSAREGPTRVARELSGHSGYLSCCRFISDKRILTSSGDMTCVLWD
Cochliobolus heterostrophus       YVACGGLDNICSIYNLSAREGPTRVARELSGHSGYLSCCRFISDKRILTSSGDMTCVLWD
Leptosphaeria maculans            YVACGGLDNICSIYNLSAREGPTRVARELSGHSGYLSCCRFISDKRILTSSGDMTCVLWD
Pyrenophora teres f.sp. teres     YVACGGLDNICSIYNLSAREGPTRVARELSGHSGYLSCCRFISDKRILTSSGDMTCVLWD
Mycosphaerella graminciola        YVACGGLDNICSIYNLSAREGPTRVARELSGHSGYLSCCRFINDRRILTSSGDMTCVLWD
                                  ****************************************** * ***************

S. nodorum                        LETGSKVHEFADHLGDVMSLSINPLDHNQFVSGACDAFAKLWDIRQQKCVQTFAAHDSDI
Cochliobolus heterostrophus       LETGSKVHEFADHLGDVMSLSINPLDHNQFVSGACDAFAKLWDIRQQKCVQTFAAHDSDI
Leptosphaeria maculans            LETGSKVHEFADHLGDVMSLSINPLDNNQFVSGACDAFAKLWDIRQQKCVQTFAAHDSDI
Pyrenophora teres f.sp. teres     LETGSKVHEFADHLGDVMSLSINPLDHNQFVSGACDAFAKLWDIRQQKCVQTFAAHDSDI
Mycosphaerella graminciola        IETGQKITEFADHLGDVMSLSINPLDNNQFVSGACDAFAKLWDIRQQKCTQTFAAHDSDI
                                  *** *  ****************** ********************** **********

S. nodorum                        NAIQFFPNGNAFGTGSDDASCRLFDIRADRELASYQIPEPVCGITSVAFSVSGRLLFAGY
Cochliobolus heterostrophus       NAIQFFPNGNAFGTGSDDASCRLFDIRADRELASYQIPEPVCGITSVAFSVSGRLLFAGY
Leptosphaeria maculans            NAIQFFPNGNAFGTGSDDASCRLFDIRADRELASYQIPEPVCGITSVAFSVSGRLLFAGY
Pyrenophora teres f.sp. teres     NAIQFFPNGNAFGTGSDDASCRLFDIRADRELASYQIPEPVCGITSVAFSVSGRLLFAGY
Mycosphaerella graminciola        NAIQFFPNGHAFGTGSDDASCRLFDIRADRELQSYTIGEPVCGITSVAFSVSGRLLFAGY
                                  ********* ********************** ** * **********************

S. nodorum                        DDFECKVWDVLRGERVGTLQGHDNRVSCLGVSNDALSLCTGSWDSMLRIWA
Cochliobolus heterostrophus       DDFECKVWDVLRGERVGTLQGHDNRVSCLGVSNDALSLCTGSWDSMLRIWA
Leptosphaeria maculans            DDFECKVWDVLRGERVGTLQGHDNRVSCLGVSNDALSLCTGSWDSMLRIWA
Pyrenophora teres f.sp. teres     DDFECKVWDVLRGERVGTLQGHDNRVSCLGVSNDALSLCTGSWDSMLRIWA
Mycosphaerella graminciola        DDFECKVWDVLRGERVGTLQGHDNRVSCLGVSNDAMSLCTGSWDSMLRIWA
                                  *********************************** ***************
```

(B)

```
S. nodorum                        MPAMMVAAPYEIRTGGDGKSKKQSMAELKLRRLTELNQRLREDLERRRIPVSEAALDLIA
Pyrenophora tritici-repentis      MSAMMTAAPYEIRTGGDGKSKKQSMAELKLRRLTELNQRLREDLERRRIPVSEAALDLIA
Mycosphaerella graminciola        -----MSSPYEIRTNENGKSKKQSMAELKLRRLTELNQRLQEDLNRRRIPVSEAALDLIA
Leptosphaeria maculans            MPAIMAAAPYEIRTGGDGKSKKQSMAELKLRRLTELNQRLREDLERRRIPVSEAALDLIA
                                       ******   *********************** *** ***************

S. nodorum                        FTDKEPKDWMVPSRWGTIDKREDPYAPQQSNGCCIVM
Pyrenophora tritici-repentis      FTDKEPKDWMVPSRWGTIDKRDDPYAPQQSNGCCIVM
Mycosphaerella graminciola        FTDKEPKDFMVPSRWGTIDKREDPYAPQQSNGCCSIM
Leptosphaeria maculans            FTDKEPKDWMVPSRWGTIDKRDDPYAPQQSSGCCIVM
                                  ******** ************ ******** ***  *
```

Figure S1 ClustalW alignment of *S. nodorum* (A) Gba1 and (B) GgaA with fungal orthologues

(A)

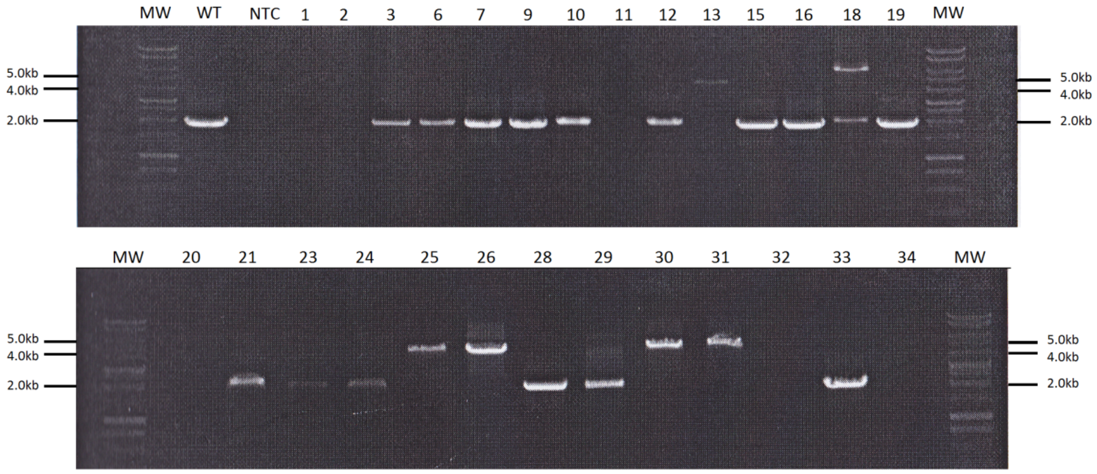

(B)

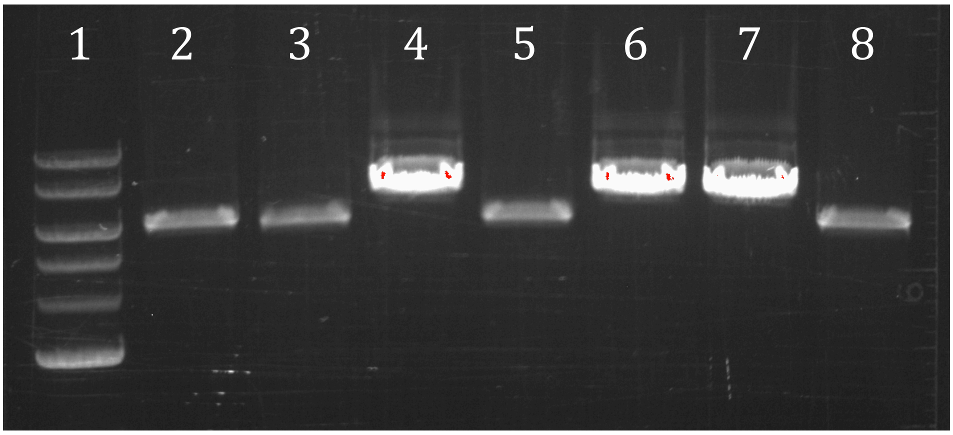

Figure S2 (A) Agarose gel electrophoresis of PCR products arising from the amplification of the (A) *GgaA* locus of the created *S. nodorum* mutants. Targeted insertion of the phleomycin cassette in place of the *S. nodorum GgaA* gene results in a 4196bp amplicon (Lanes 25, 26, 30, 31) , replacing the 1789bp amplicon of the wild type (WT) SN15. MW, Molecular weight marker; WT, *S. nodorum* SN15 gDNA; NTC, no template PCR control; the remaining lanes labeled by mutant culture number. Lanes 1, 2, 11, 20, 32, 34, no observed amplification or (B) *Gba1* locus of strains transformed with the *Gba1* homologous disruption construct. A band of 6.1kb represents the wildtype locus and 7.6kb the locus having undergone homologous recombination with the disruption construct. Lane 1, 1kb ladder; Lane 2, *S. nodorum* SN15 (wildtype); Lanes 3-8, a representative selection of transformants. Strains represented in lanes 4, 6 and 7 have all undergone homologous recombination and represent *Gba1* mutants.

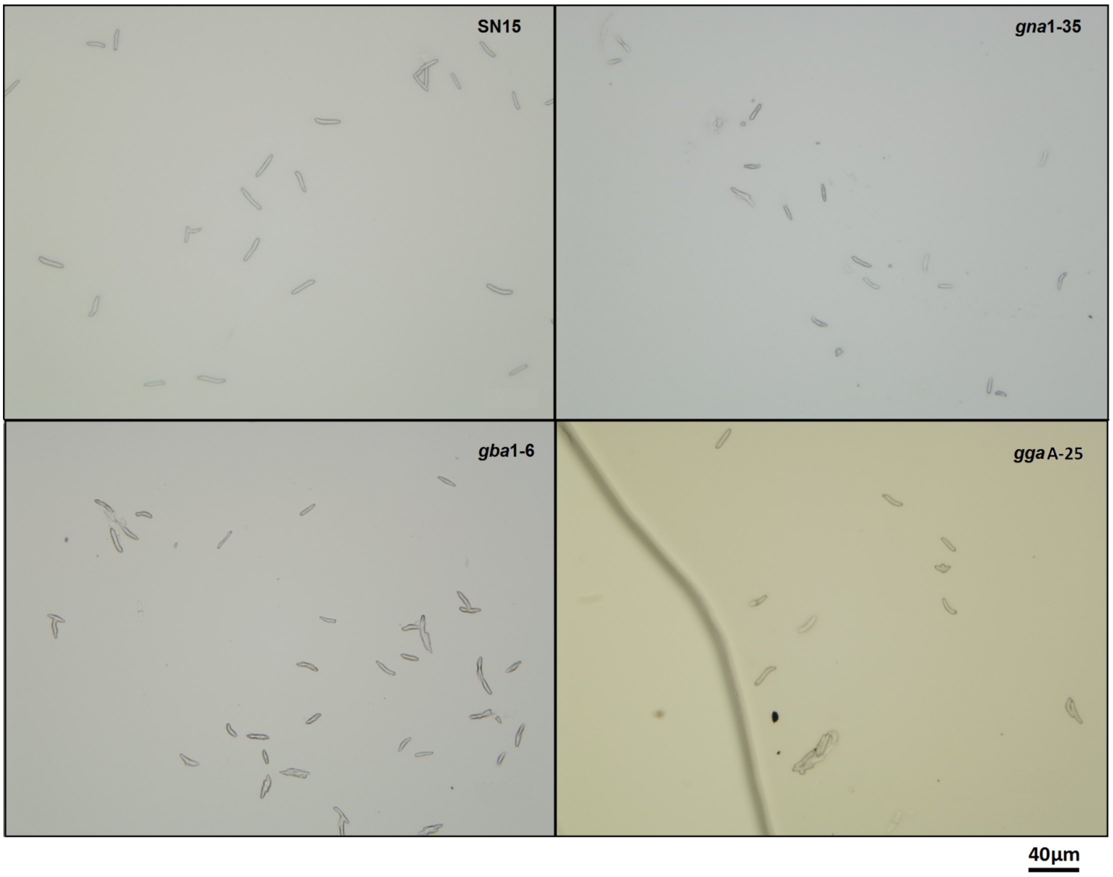

Figure S3 Light microscopy of the asexual spores of *S. nodorum*, harvested from the wild-type SN15 and mutant strains *gna*1-35, *gba*1-6 and *gga*A-25.
