# Supplementary material for: A comparative analysis of the heterotrimeric G-protein Gα, Gβ and Gγ subunits in the wheat pathogen Stagonospora nodorum
Source: BMC Microbiol. 2012 Jul 3;12:131. doi: 10.1186/1471-2180-12-131 (PMC3492189; doi:10.1186/1471-2180-12-131)
Supplement: Additional file 2 — Table S1. Sequences of primers used in this study. [file 1471-2180-12-131-S2.docx]

**Supplementary Table S1 Sequences of primers used in this study.**

| ***Primer name*** | ***Sequence*** |
| --- | --- |
| PHLprimer | GCTGATGAACAGGGTCAC |
| M13 R-primer | GGAAACAGCTATGACCCATG |
| LEOprimer | AGTTGACCAGTGCCGTTC |
| M13-Fprimer | GTAAAACGACGGCCAGTG |
| GgaA5'Fprimer | GCAAGGCTGCTGCTACTATAGT |
| GgaA3'Fprimer | CATGGTCATAGCTGTTTCCGCCAATGCTAACATGACC |
| GgaA5'Rprimer | CACTGGCCGTCGTTTTACCATGCTCTGCTTCTTGCTCT |
| GgaA3'Rprimer | GGGTCAAAATACGTCTTCC |
| 00288KO5f | CTGGTGTTGTCACGCAAGT |
| 00288KO3r | ATCCATGTGCAAGCCTTG |
| GbaKO5’f | AGGGTACCATGGCCGATATGAATCAAGA |
| GbaKO5’r | TGCCTGCAGTAAGCGCATCCTCACCTCGT |
| GbaKO3’f | ACTCGAGTACAGCAACTGAGGTAGCCG |
| GbaKO3’r | TGGATCCCAAAGACTGAGCGCGTCATT |
| GbaKOscreenF | GGAGACGAGATGTCTGCTGT |
| GbaKOScreenR | ATGCGACGCCATGTGACGAT |
| GgaKOscreenF | CTGGTGTTGTCACGCAAGT |
| GgaKOscreenR | ATCCATGTGCAAGCCTTG |
| ActinqPCRf | AGTCGAAGCGTGGTATCCT |
| ActinqPCRr | ACTTGGGGTTGATGGAG |
| PhleoqPCRf | ACTTCATCGCAGCTTGACTAAC |
| PhleoqPCRr | TGATGAACAGGGTCACGTC |
